# Alcohol industry’s arguments against French pregnancy warning labels: a press coverage analysis

**DOI:** 10.1093/eurpub/ckac131.534

**Published:** 2022-10-25

**Authors:** A Millot, M Serra, K Gallopel-Morvan

**Affiliations:** 1 Arènes UMR CNRS 6051, Inserm U1309, Ecole des Hautes Etudes en Santé Publique, Rennes, France; 2 Département des Sciences en Santé Environneme, Ecole des Hautes Etudes en Santé Publique, Rennes, France

## Abstract

**Background:**

Alcohol drinking during pregnancy has harmful consequences. Warnings displayed on alcohol bottles are an effective measure to inform people about these risks and have been put in place in France. However, the alcohol industry (AI) resisted this measure when it was introduced in 2007 and during an expansion project in 2018. This study aims to identify arguments used by the AI against warnings targeting pregnant women.

**Methods:**

A documentary method was used to analyse these arguments disseminated by the AI and its partners (elected representatives of wine-producing regions, etc.) in the French mainstream press (the national, regional and specialised press) from 2000 to 2020 through the Europresse documentary database. A quantitative analysis (number and evolution of press articles, mapping of the actors of AI who expressed themselves) and an inductive thematic content analysis (analytical framework of the arguments identified) using NVivo Software were carried out.

**Results:**

Among the 85 articles included in this study, a majority of the arguments used by the AI are against this measure. It argues that this measure (1) is a questionable measure because ineffective in changing behaviours, (2) will have counterproductive effects (on women and on the economy); and (3) there are other preferred alternatives than warnings (targeted prevention programs, etc.). A minority is nevertheless in favour of this measure. Among the actors who expressed themselves, a large majority comes from the winegrowing sector.

**Conclusions:**

The analysis of these arguments will add new insights about AI lobbying against warnings, by analyzing the arguments over a 20 years period covering a failure and a success of industry lobbying. It will also be useful for public health advocacy to better counter this lobbying influence and these arguments, which are not necessarily evidence based.

**Key messages:**

• Warnings displayed on alcohol bottles are an effective measure which is challenged by the alcohol industry.

• Analysing the arguments disseminated by the alcohol industry is useful for public health advocacy in order to counter them.

